# Circ_0068087 Silencing Ameliorates Oxidized Low-Density Lipoprotein-Induced Dysfunction in Vascular Endothelial Cells Depending on miR-186-5p-Mediated Regulation of Roundabout Guidance Receptor 1

**DOI:** 10.3389/fcvm.2021.650374

**Published:** 2021-05-26

**Authors:** Shuanghong Li, Tao Huang, Limin Qin, Luchang Yin

**Affiliations:** ^1^Department of Emergency, Weifang Hospital of Traditional Chinese Medicine, Weifang, China; ^2^Department of Cardiovascular Medicine, Affiliated Hospital of Weifang Medical University, Weifang, China

**Keywords:** atherosclerosis, ox-LDL, circ_0068087, miR-186-5p, ROBO1

## Abstract

**Background:** Circular RNAs (circRNAs) are endogenous non-coding RNAs involved in the progression of atherosclerosis (AS). We investigated the role of circ_0068087 in AS progression and its associated mechanism.

**Methods:** The 3-(4,5-Dimethylthiazol-2-yl)-2,5-Diphenyltetrazolium Bromide (MTT) assay, flow cytometry, and enzyme-linked immunosorbent assay (ELISA) were performed to analyze the viability, apoptosis, and inflammatory response of HUVECs, respectively. Reverse transcription-quantitative polymerase chain reaction (RT-qPCR) and the Western blot assay were performed to measure the expression of RNA and protein. Cell oxidative stress was analyzed using commercial kits. The dual-luciferase reporter assay and RNA immunoprecipitation (RIP) assay were conducted to verify the interaction between microRNA-186-5p (miR-186-5p) and circ_0068087 or roundabout guidance receptor 1 (ROBO1).

**Results:** Oxidized low-density lipoprotein (ox-LDL) exposure upregulated the circ_0068087 level in HUVECs. ox-LDL-induced dysfunction in HUVECs was largely attenuated by the silence of circ_0068087. Circ_0068087 negatively regulated the miR-186-5p level by interacting with it in HUVECs. Circ_0068087 knockdown restrained ox-LDL-induced injury in HUVECs partly by upregulating miR-186-5p. ROBO1 was a downstream target of miR-186-5p in HUVECs. Circ_0068087 positively regulated ROBO1 expression by sponging miR-186-5p in HUVECs. MiR-186-5p overexpression exerted a protective role in ox-LDL-induced HUVECs partly by downregulating ROBO1.

**Conclusion:** Circ_0068087 interference alleviated ox-LDL-induced dysfunction in HUVECs partly by reducing ROBO1 expression via upregulating miR-186-5p.

## Introduction

Atherosclerosis (AS) is a chronic inflammatory disorder that contributes to the progression of various cardiovascular diseases (CVDs). AS is one of the causes of the high mortality in the aged population (1). There are three mainstream theories regarding the pathogenesis of AS, including the lipid infiltration theory, endothelial injury theory, and vascular smooth muscle cell migration/proliferation theory (2). Endothelial cells are widely distributed in vascular networks, and endothelial dysfunction is identified as a vital factor in AS pathogenesis (3). Oxidized low-density lipoprotein (ox-LDL) is a critical inducer for the dysfunction of endothelial cells (4, 5). In this study, we explored the mechanism underlying the aberrant transformation of endothelial cells in ox-LDL-induced AS cell model.

Circular RNAs (circRNAs) possess a covalently closed loop structure produced by the back-splicing of premessenger RNAs (pre-mRNAs) (6). The aberrant expression of circRNAs has been associated with the progression of CVD (7). Zhang et al. find that circ_0003204 blocks the proliferation ability, migration capacity, and angiogenesis of endothelial cells in AS (8). Yang et al. demonstrate that circ-CHFR enhances the proliferation and migration abilities of vascular smooth muscle cells by regulating the microRNA-370 (miR-370)/FOXO1/Cyclin D1 signaling (9). Circ_0068087 is derived from the back-splicing of partial fragments in the GNB4 gene. Cheng et al. demonstrate that circ_0068087 aggravates high glucose–induced dysfunction and inflammation of endothelial cells by sponging miR-197 in diabetes mellitus (10). However, the role of circ_0068087 in AS has never been illustrated.

MicroRNAs (miRNAs) induce mRNA degradation or translational inhibition through interacting with the 3'-untranslated regions (3'UTRs) of mRNAs (11). MiRNAs are vital regulators in AS pathogenesis. For instance, miR-210-3p alleviates lipid accumulation and inflammatory response by suppressing the level of IGF2 in AS (12). Based on bioinformatic analysis, miR-186-5p is predicted as a possible target of circ_0068087. Dang et al. find that circ_0010729 promotes the proliferation and migration and suppresses the apoptosis of HUVECs by targeting the miR-186/HIF-1α axis (13). Nevertheless, the functional association between circ_0068087 and miR-186-5p in AS remains unclear.

Roundabout guidance receptor 1 (ROBO1; Accession number: NM_002941.4) is the receptor of slit guidance ligand 1 (SLIT1) and SLIT2. ROBO1 is identified as an oncogene in human malignancies. For instance, miR-218 suppresses the motility of lung cancer cells by downregulating ROBO1 (14). Liu et al. find that ROBO1 is a target of miR-29a, and miR-29a restrains the motility of gastric cancer cells through repressing the expression of ROBO1 (15). Pan et al. find that high glucose treatment upregulates the level of ROBO1 (16). However, the role of ROBO1 in AS progression is barely known.

The expression and function of circ_0068087 in ox-LDL-induced endothelial cells were explored. The potential regulatory mechanism behind circ_0068087 was investigated using bioinformatic databases and rescue experiments.

## Materials and Methods

### Cell Cultivation

Human umbilical vein endothelial cells (HUVECs) were obtained from the Chinese Academy of Medical Sciences, Shanghai Institute Cell Bank (Shanghai, China). HUVECs were cultivated using Dulbecco's modified Eagle's medium (Invitrogen, Waltham, MA, USA) supplemented with 10% fetal bovine serum (FBS, Sigma, St. Louis, MO, USA) and 1% antibiotic mixture (Sangon Biotech, Shanghai, China) at 37°C with 5% CO_2_. HUVECs at passages 3–8 were utilized in all experiments. The utilization of HUVECs has gotten the permission of the Ethics Committee of Weifang Hospital of Traditional Chinese Medicine.

### Establishment of as Cell Model

The AS cell model was established through exposing HUVECs to 40 mg/L ox-LDL (Solarbio, Beijing, China) for 24 h.

### 3-(4,5-Dimethylthiazol-2-yl)-2,5-Diphenyltetrazolium Bromide (MTT) Assay

Cell viability was analyzed by MTT assay. Transfected HUVECs in 96-well plates were incubated with 20 μL MTT reagent (Sigma), and the culture supernatant was discarded 2 h later. A total of 150 μL dimethylsulfoxide (DMSO; Sigma) solution was added into each well, and cell culture plates were shaken for 0.5 h. The optical density at 570 nm was determined using the microplate reader (Bio-Rad, Hercules, CA, USA).

### Reverse Transcription-Quantitative Polymerase Chain Reaction

RNA samples were isolated using Trizol reagent (Invitrogen). Isolated RNAs were used to synthesize complementary DNA (cDNA) using a MicroRNA Reverse Transcription Kit (Applied Biosystems, Foster City, CA, USA) and TaqMan Reverse Transcription Reagents (Invitrogen). qPCR reaction was performed using a mirVana^TM^ RT-qPCR miRNA Detection Kit (Ambion, Austin, TX, USA) and SYBR^TM^ Green PCR Master Mix (Invitrogen). The relative expression levels were calculated by the 2^−ΔΔCt^ method. Glyceraldehyde-3-phosphate dehydrogenase (GAPDH) served as the internal reference for circ_0068087 and ROBO1, and U6 functioned as the reference for miR-186-5p. The primer sequences are shown in Table 1.

**Table 1:** Primer sequences in RT-qPCR assay.

**Gene**	**Primer (5^**′**^-3^**′**^)**
circ_0068087	CAGTGGCTTTATGAAATGTTGTG (forward; F)
	CTAGGTGGCCCCTCAGTGTA (reverse; R)
miR-186-5p	CAAAGAATTCTCCTTT (F)
	GAACATGTCTGCGTATCTC (R)
ROBO1	GGAAGAAGACGAAGCCGACAT (F)
	TCTCCAGGTCCCCAACACTG (R)
U6	CTCGCTTCGGCAGCACA (F)
	AACGCTTCACGAATTTGCGT (R)
GAPDH	TTCACCACCATGGAGAAGGC (F)
	GGCCAGGGGTGCTAAGCAGT (R)

### Subcellular Fractionation Location

The cytoplasmic and nuclear RNAs were extracted using the PARIS^TM^ Kit Protein and RNA Isolation system (Thermo Fisher Scientific, Waltham, MA, USA) in accordance with the manufacturer's instructions.

### Transient Transfection

To silence circ_0068087, small interfering (si)RNA targeting circ_0068087 (si-circ_0068087; 5'-GTGCTTTTAACCAGAATTACA-3') was synthesized to target the junction sites of circ_0068087, and negative control of siRNA (si-NC; 5'-GTGTGCAGTAGCAGTAA-3') was utilized as the control. MiR-186-5p mimic (5'-TCGGGTTTTCCTCTTAAGAAAC-3') or miR-186-5p inhibitor (5'-ACGTAGGACTGGACAAAC-3') was synthesized to upregulate or silence miR-186-5p, and miRNA NC (5'-CTGATTAGCATACAGTGG-3') and inhibitor NC (5'-GTGCGTAGGCATTACAGTA-3') were utilized as the controls. To upregulate ROBO1, the ectopic expression plasmid of ROBO1 using pcDNA vector (pc-ROBO1) was constructed, and pc-NC was utilized as the control. RNAs and plasmids provided by GenePharma (Shanghai, china) and Sangon Biotech were transfected into HUVECs using the Lipofectamine 3000 (Invitrogen) reagent when cell confluence reached about 70%.

### Flow Cytometry

HUVECs were collected and resuspended in 200 μL binding buffer (Qiagen, Valencia, CA, USA). Afterward, Annexin V-fluorescein isothiocyanate (Annexin V-FITC; Qiagen) and propidium iodide (PI; Qiagen) were pipetted to incubate with HUVECs. The apoptosis rate of HUVECs was analyzed by the BD FACSCalibur flow cytometer (BD Biosciences, Franklin Lakes, NJ, USA).

### Western Blot Assay

HUVECs were washed using ice-cold phosphate buffer saline (PBS; Sangon Biotech) and disrupted using whole cell lysate buffer (Beyotime, Shanghai, China). Equal amounts of protein samples were electrophoretically separated on sodium dodecyl sulfate-polyacrylamide gel electrophoresis (SDS-PAGE) and were transferred onto a polyvinylidene difluoride (PVDF) membrane (Millipore, Billerica, MA, USA). Afterward, the membrane was blocked through incubating using 5% milk and was incubated with the primary antibodies overnight. Primary antibodies including antiproliferating cell nuclear antigen (anti-PCNA; ab92552), anti-B cell leukemia/lymphoma 2 (anti-Bcl-2; ab182858), anti-ROBO1 (ab7279), anti-Ki67 (ab16667), anti-Bcl-2 associated X, apoptosis regulator (anti-Bax; ab32503), and anti-GAPDH (ab8245), were purchased from Abcam (Cambridge, MA, USA). The secondary antibody (Abcam; ab205718) labeled with horseradish peroxidase was then incubated with the membrane for 2 h. The protein signals were visualized using an enhanced chemiluminescent (ECL) chromogenic substrate (Beyotime).

### Enzyme-Linked Immunosorbent Assay

The levels of interleukin 6 (IL-6; D6050), IL-1β (201-LB) and tumor necrosis factor α (TNF-α; MTA00B) in the culture supernatant were analyzed using the Human IL-6/IL-1β/TNF-α Quantikine ELISA Kit (R&D Systems, Minneapolis, MN, USA) according to the manufacturer's instructions.

### Detection of Malondialdehyde Level, Reactive Oxygen Species Level, and Superoxide Dismutase Activity

The levels of MDA (A003-4) and ROS (E004) and the activity of SOD (A001-3) were analyzed by their corresponding kits (Jiancheng Biotech, Nanjing, China) in accordance with the manufacturer's instructions.

### Establishment of circRNA/miRNA/mRNA Axis

Bioinformatic database circinteractome (https://circinteractome.irp.nia.nih.gov) was utilized to predict the circ_0068087-miRNAs interactions, and the StarBase database (http://starbase.sysu.edu.cn) was utilized to predict the miR-186-5p/mRNA interactions.

### Dual-Luciferase Reporter Assay

The WT-circ_0068087 and WT-ROBO1-3'UTR reporter plasmids were constructed through inserting the partial fragment of circ_0068087 or ROBO1 3'UTR into the psiCHECK2 (Promega, Madison, WI, USA) vector. The GeneArt^TM^ Site-Directed Mutagenesis System (Invitrogen) was utilized to amplify the mutant fragment. The mutant fragment of circ_0068087 or ROBO1 3'UTR, including the mutant binding sites with miR-186-5p, was also inserted into the psiCHECK2 vector (Promega) to generate MUT-circ_0068087 and MUT-ROBO1-3'UTR. HUVECs were cotransfected with luciferase plasmids and miR-186-5p mimic or miRNA NC. The luciferase intensity was determined using a commercial Dual-Luciferase Reporter Assay Kit (Promega).

### RNA Immunoprecipitation Assay

The Magna RIP^TM^ RNA-Binding Protein Immunoprecipitation Kit (Millipore) was utilized to test whether miR-186-5p was a target of circ_0068087 in HUVECs. HUVECs were disrupted in RIP lysis buffer, and cell extracts were mixed with RIP buffer containing Argonaute2 (Ago2) antibody (Abcam, ab186733)- or immunoglobulin G (IgG) antibody (Abcam, ab172730)-precoated magnetic beads. RNA enrichment was determined by RT-qPCR.

### Statistical Analysis

Data were analyzed using GraphPad Prism 7.0 software (GraphPad, La Jolla, CA, USA) and were expressed in the form of mean ± standard deviation (SD). The unpaired Student *t*-test was utilized to assess the differences in two groups. The differences in multiple groups were assessed using the one-way analysis of variance (ANOVA) followed by Tukey's *post hoc* test. *P* < 0.05 was considered statistically significant.

## Results

### Circ_0068087 Is Upregulated by ox-LDL in HUVECs

**Figure 1:**
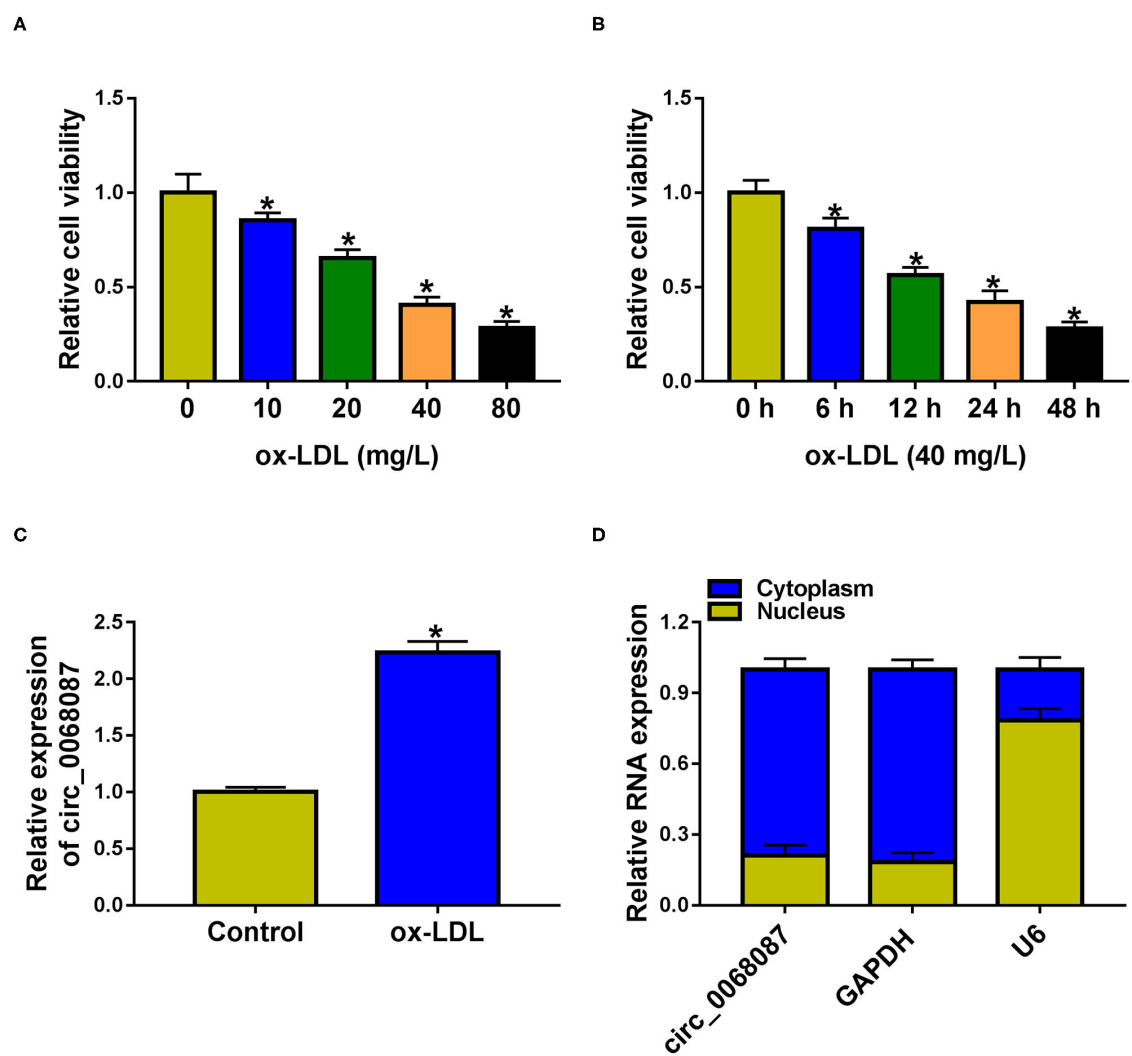
Circ_0068087 is upregulated by ox-LDL in HUVECs. **(A)** Cell viability in HUVECs exposed to different doses of ox-LDL (10, 20, 40, and 80 mg/L) for 24 h was determined by MTT assay. This experiment was performed three times with five technical repetitions each time. One-way ANOVA followed by Tukey's *post hoc* test was used to assess the differences. **(B)** MTT assay was utilized to analyze cell viability of HUVECs stimulated by 40 mg/L ox-LDL for 6, 12, 24, or 48 h. This experiment was performed three times with five technical repetitions each time. One-way ANOVA followed by Tukey's *post hoc* test was used to assess the differences. **(C)** The expression of circ_0068087 was examined in HUVECs treated with or without 40 mg/L ox-LDL for 24 h by RT-qPCR. This experiment was performed three times with three technical repetitions each time. An unpaired Student *t*-test was used to evaluate the differences. **(D)** The subcellular location of circ_0068087 in HUVECs was analyzed. This experiment was performed three times. ^*^P < 0.05.

We find that ox-LDL exposure reduced the viability of HUVECs in a dose- and time-dependent manner (Figures 1A,B). ox-LDL (40 mg/L; 24 h) was chosen for further experiments. ox-LDL treatment significantly upregulated circ_0068087 expression in HUVECs (Figure 1C). We assessed the subcellular localization of circ_0068087 in HUVECs prior to exploring its biological function. Circ_0068087 was mainly localized in the cytoplasmic fraction of HUVECs (Figure 1D), which endowed circ_0068087 the potential to serve as a miRNA sponge.

### ox-LDL Suppresses the Viability and Induces the Apoptosis, Inflammation, and Oxidative Stress of HUVECs Partly Through Upregulating Circ_0068087

**Figure 2:**
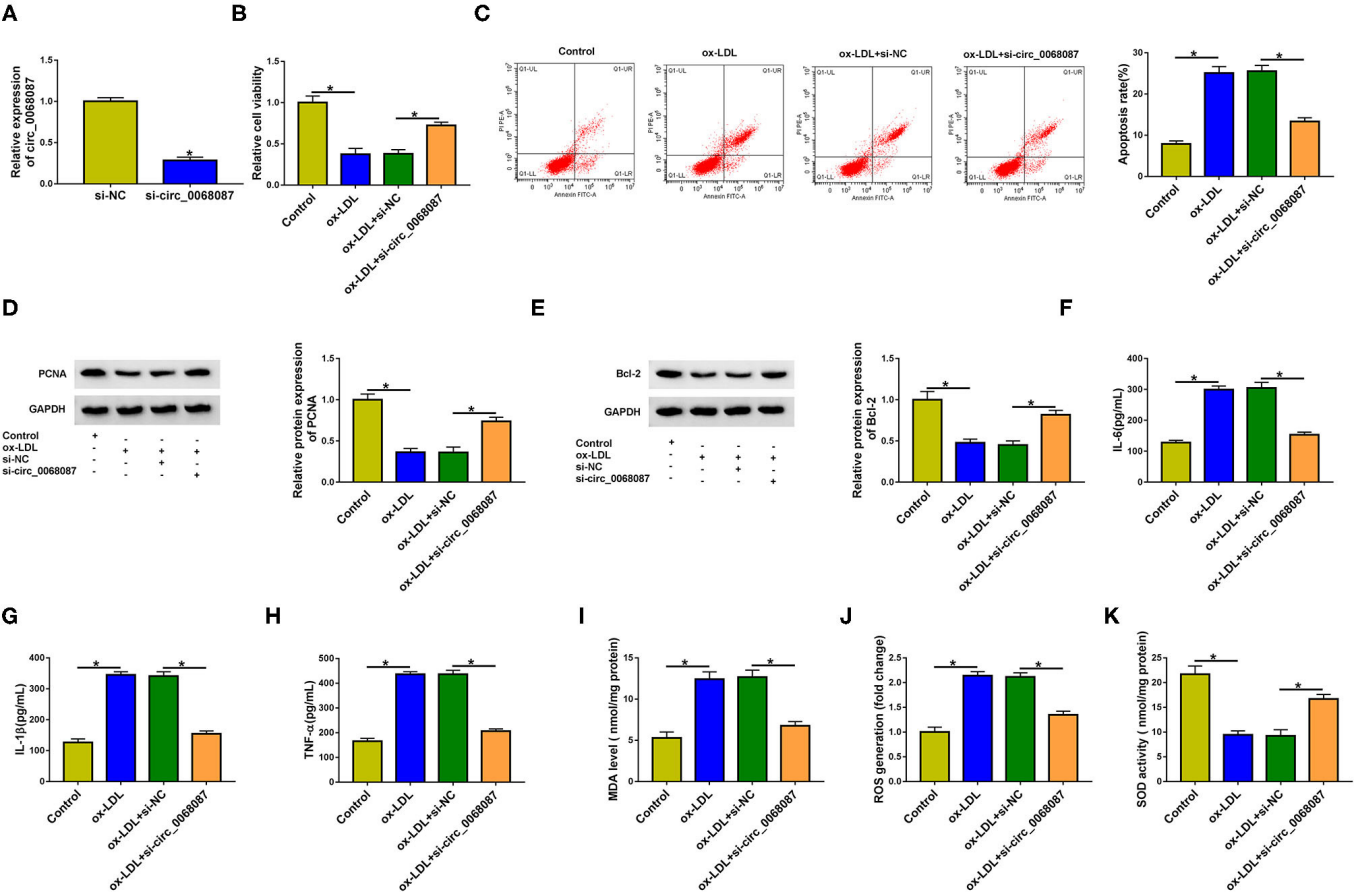
Ox-LDL suppresses the viability and induces the apoptosis, inflammation, and oxidative stress of HUVECs partly through upregulating circ_0068087. **(A)** The interference efficiency of circ_0068087 siRNA (si-circ_0068087) in HUVECs was determined by RT-qPCR. This experiment was performed three times with three technical repetitions each time. An unpaired Student *t*-test was used to evaluate the differences. **(B–K)** HUVECs were divided into the following four groups: Control, ox-LDL, ox-LDL + si-NC, and ox-LDL + si-circ_0068087. One-way ANOVA followed by Tukey's *post hoc* test was used to assess the differences. **(B)** Cell viability was analyzed by MTT assay. This experiment was performed three times with five technical repetitions each time. **(C)** The apoptosis rate of HUVECs was analyzed by flow cytometry. This experiment was performed three times with three technical repetitions each time. **(D,E)** Western blot assay was performed to measure the protein expression of PCNA and Bcl-2 in HUVECs. This experiment was performed three times. **(F–H)** The concentrations of pro-inflammatory cytokines (IL-6, IL-1β, and TNF-α) in the culture supernatant were measured by ELISA. This experiment was performed three times with three technical repetitions each time. **(I–K)** Three oxidative stress-related markers (MDA, ROS, and SOD) were determined by commercial kits. This experiment was performed three times with three technical repetitions each time. *P < 0.05.

To explore whether ox-LDL-induced effects on HUVECs were partly attributed to the upregulation of circ_0068087, HUVECs were transfected with si-circ_0068087 prior to ox-LDL exposure. High transfection efficiency of si-circ_0068087 was confirmed via RT-qPCR assay in HUVECs (Figure 2A). ox-LDL exposure suppressed cell viability, which was largely counteracted by circ_0068087 silencing in HUVECs (Figure 2B). Cell apoptosis was triggered by ox-LDL stimulation, and the interference of circ_0068087 restrained the apoptosis of ox-LDL-induced HUVECs (Figure 2C). To further explore whether ox-LDL restrained cell viability and induced cell apoptosis via upregulating circ_0068087, we measured the protein levels of proliferation markers (PCNA and Ki67), anti-apoptotic protein (Bcl-2), and pro-apoptotic protein (Bax). Circ_0068087 silencing rescued the levels of PCNA, Ki67, and Bcl-2 and decreased the expression of Bax in ox-LDL-induced HUVECs (Figures 2D,E, Supplementary Figure 1A). ox-LDL exposure induced the release of inflammatory cytokines (IL-6, IL-1β, and TNF-α), and circ_0068087 silencing largely attenuated ox-LDL-induced influences (Figures 2F–H), demonstrating that ox-LDL-induced inflammation of HUVECs was partly based on the upregulation of circ_0068087. Oxidative stress contributes to the progression of AS. Vascular oxidative stress induces multiple molecular events in AS progression, including oxidative modification of lipoproteins and phospholipids, macrophage infiltration, and foam cell formation (17). We determined the levels or activity of oxidative stress-associated markers (MDA, ROS, and SOD) to analyze cellular oxidative stress status. ox-LDL exposure increased the levels of MDA and ROS, whereas it inhibited the activity of SOD, and these effects were largely counteracted by the silence of circ_0068087 in HUVECs (Figures 2I–K). These findings suggest that ox-LDL-induced dysfunction in HUVECs was partly dependent on the upregulation of circ_0068087.

### Circ_0068087 Acts as an miR-186-5p Sponge in HUVECs

**Figure 3:**
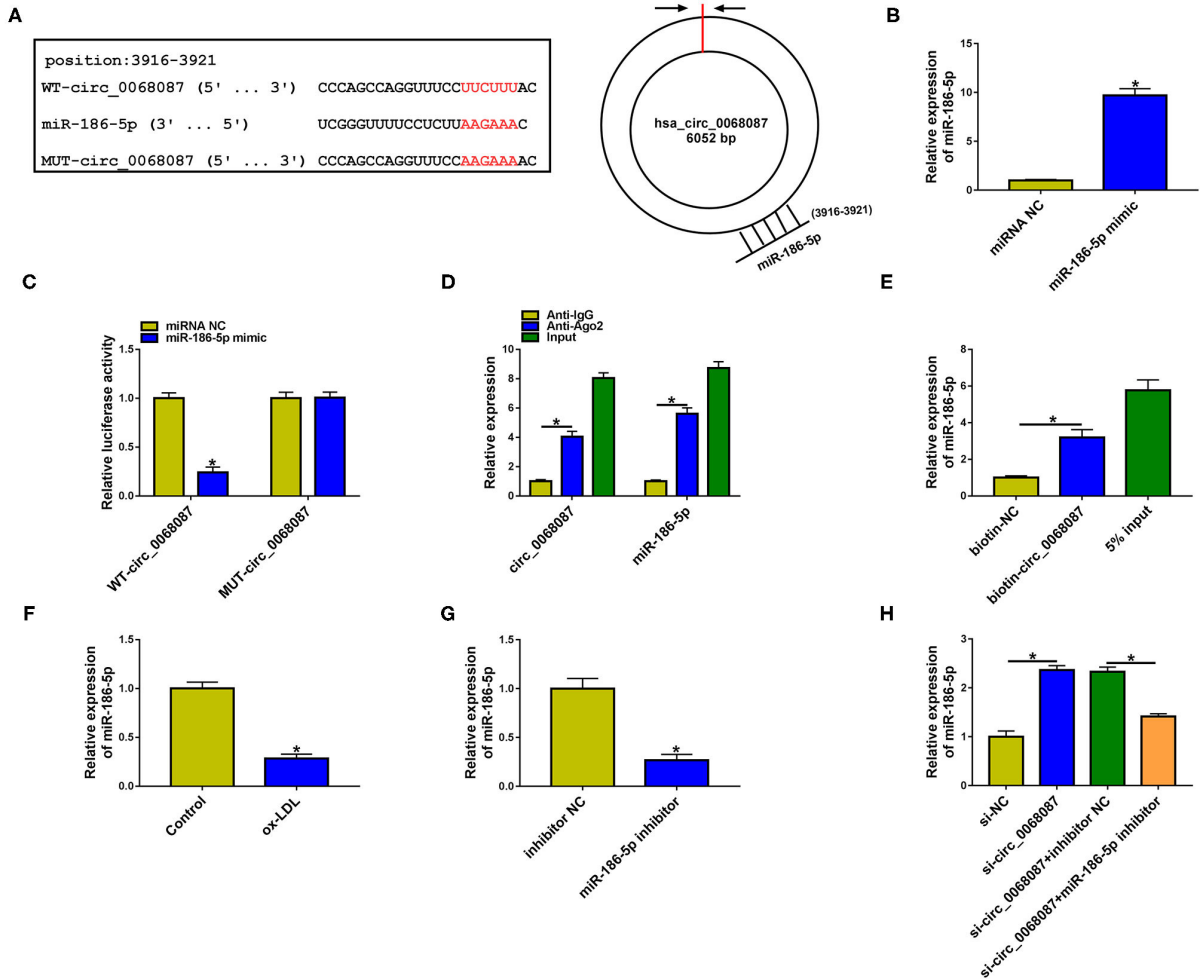
Circ_0068087 acts as miR-186-5p sponge in HUVECs. **(A)** The putative binding sites between circ_0068087 and miR-186-5p were predicted by circinteractome and circBank. **(B)** The overexpression efficiency of miR-186-5p mimic in HUVECs was assessed by RT-qPCR. This experiment was performed three times with three technical repetitions each time. An unpaired Student *t*-test was used to evaluate the differences. **(C,D)** The predicted interaction between circ_0068087 and miR-186-5p was tested by dual-luciferase reporter assay and RIP assay. These two experiments were performed three times with three technical repetitions each time. An unpaired Student *t*-test was used to evaluate the differences. **(E)** RNA pulldown assay was applied to test the interaction between circ_0068087 and miR-186-5p. This experiment was performed three times with three technical repetitions each time. An unpaired Student *t*-test was used to evaluate the differences. **(F)** The level of miR-186-5p was measured in HUVECs exposed to 40 mg/L ox-LDL for 24 h by RT-qPCR. This experiment was performed three times with three technical repetitions each time. An unpaired Student *t*-test was used to evaluate the differences. **(G)** The silence efficiency of miR-186-5p inhibitor in HUVECs was assessed by RT-qPCR. This experiment was performed three times with three technical repetitions each time. An unpaired Student *t*-test was used to evaluate the differences. **(H)** The expression of miR-186-5p in HUVECs transfected with si-NC, si-circ_0068087, si-circ_0068087 + inhibitor NC, and si-circ_0068087 + miR-186-5p inhibitor was tested by RT-qPCR. This experiment was performed three times with three technical repetitions each time. One-way ANOVA followed by Tukey's *post hoc* test was used to assess the differences. *P < 0.05.

CircRNAs have shown their “miRNA sponge” role in regulating cellular physiological and pathological processes (18, 19). We performed bioinformatic analysis using circinteractome and circbank databases to predict the possible targets of circ_0068087. There were 43 candidate miRNA targets of circ_0068087 predicted by both databases (Supplementary Figure 2A). Among these miRNAs, we screened out five miRNAs that were implicated in AS progression, including miR-186-5p (13), miR-140-3p (20), miR-640 (21), miR-515-5p (22), and miR-665 (23). MiR-186-5p was selected for further experiments due to it having the most significant negative regulatory relationship with circ_0068087 (Supplementary Figure 2A). The putative binding sites with miR-186-5p in circ_0068087 (3916 bp-3921 bp) are shown in Figure 3A. RT-qPCR verified the high overexpression efficiency of miR-186-5p mimic in HUVECs (Figure 3B). Transfection with miR-186-5p mimic significantly reduced the luciferase activity of wild-type luciferase reporter plasmid (WT-circ_0068087) rather than its mutant plasmid (MUT-circ_0068087) (Figure 3C), suggesting that miR-186-5p was a target of circ_0068087 in HUVECs. The RIP assay was employed to further confirm the target relationship between circ_0068087 and miR-186-5p. MiRNAs can form the ribonucleoprotein complexes (miRNPs) that include Ago2, the core component of the RNA-induced silencing complex (RISC) (24). As shown in Figure 3D, circ_0068087 and miR-186-5p were both enriched in the Ago2 antibody group. MiR-186-5p was enriched when using biotinylated circ_0068087 (biotin-circ_0068087) (Figure 3E). The results of the RIP assay and RNA pulldown assay further validated the interaction between circ_0068087 and miR-186-5p. ox-LDL exposure downregulated the expression of miR-186-5p in HUVECs (Figure 3F). The results of RT-qPCR confirmed the high knockdown efficiency of the miR-186-5p inhibitor in HUVECs (Figure 3G). Circ_0068087 silencing upregulated the expression of miR-186-5p, and the expression of miR-186-5p was reduced by the introduction of the miR-186-5p inhibitor in HUVECs (Figure 3H), demonstrating the negative regulatory relation between circ_0068087 and miR-186-5p. These results suggest that circ_0068087 negatively regulates miR-186-5p expression by sponging it in HUVECs.

### Circ_0068087 Silencing Attenuates ox-LDL-Induced Damage in HUVECs Partly by Upregulating miR-186-5p

**Figure 4:**
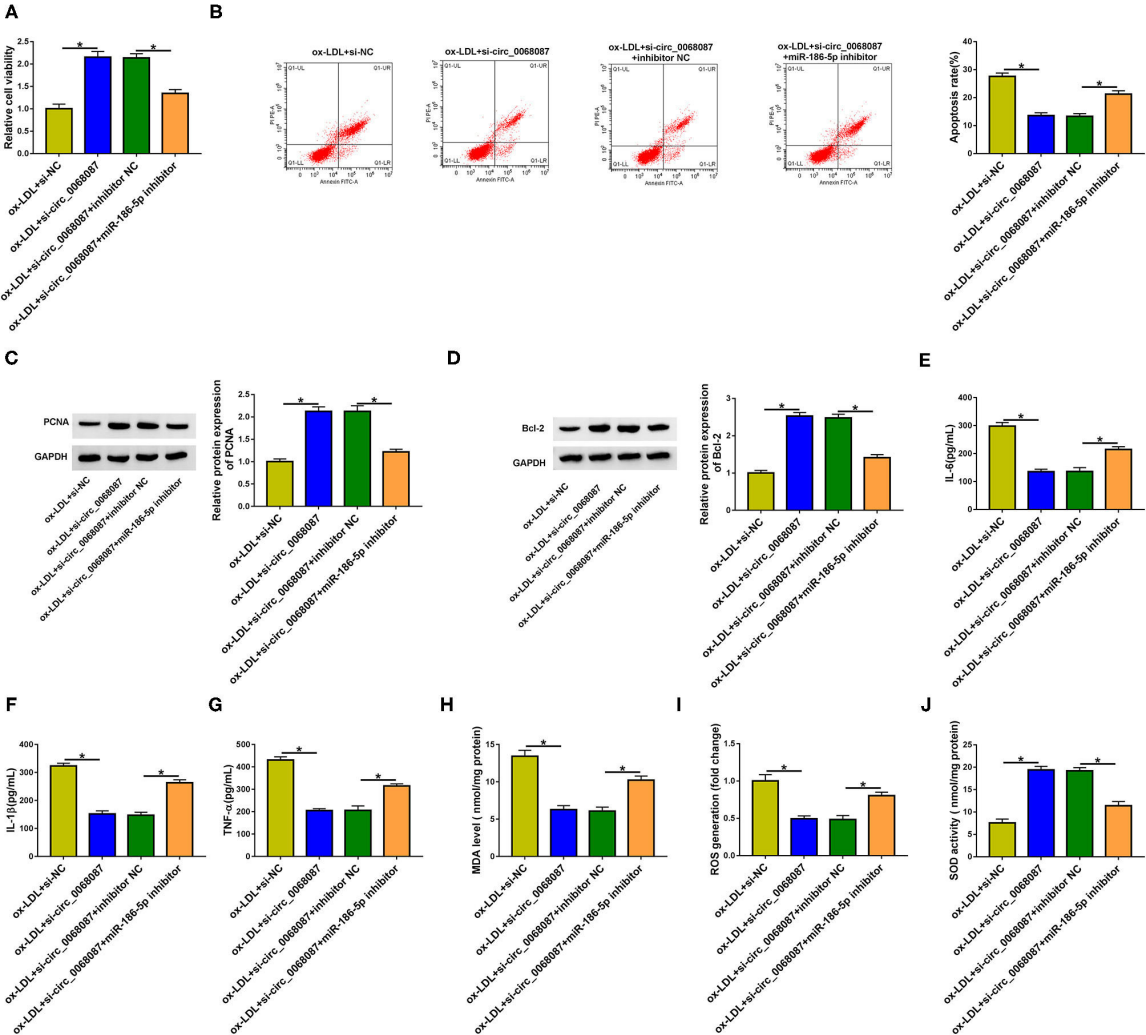
Circ_0068087 silencing attenuates ox-LDL-induced damage in HUVECs partly by upregulating miR-186-5p. **(A–J)** HUVECs were transfected with si-circ_0068087 alone or together with miR-186-5p inhibitor prior to ox-LDL exposure (40 mg/L; 24 h). One-way ANOVA followed by Tukey's *post hoc* test was used to assess the differences. **(A)** MTT assay was performed to analyze cell viability. This experiment was performed three times with five technical repetitions each time. **(B)** Cell apoptosis rate was assessed through performing flow cytometry. This experiment was performed three times with three technical repetitions each time. **(C,D)** The levels of proliferation marker (PCNA) and apoptosis marker (Bcl-2) were measured by Western blot assay. This experiment was performed three times. **(E–G)** Cell inflammatory response was evaluated by ELISA. This experiment was performed three times with three technical repetitions each time. **(H–J)** The levels of MDA and ROS and the activity of SOD were determined by their commercial kits. This experiment was performed three times with three technical repetitions each time. *P < 0.05.

Rescue experiments were performed through transfecting HUVECs with si-circ_0068087 alone or together with an miR-186-5p inhibitor prior to ox-LDL exposure. MiR-186-5p knockdown suppressed cell viability in circ_0068087-silenced HUVECs upon ox-LDL exposure (Figure 4A). MiR-186-5p silencing triggered cell apoptosis in circ_0068087-silenced HUVECs again upon ox-LDL treatment (Figure 4B). Circ_0068087 silencing mediated the promoting effect on the expression of PCNA, Ki67, and Bcl-2, and the suppressive effects on the level of Bax were largely reversed by the introduction of the miR-186-5p inhibitor in ox-LDL-induced HUVECs (Figures 4C,D, Supplementary Figure 1B). These results demonstrate that circ_0068087 knockdown attenuates ox-LDL-induced effects on the viability and apoptosis of HUVECs largely by upregulating miR-186-5p. Circ_0068087 silencing suppressed the cell inflammatory response, which was largely counteracted by the knockdown of miR-186-5p (Figures 4E–G). Circ_0068087 knockdown decreased the levels of MDA and ROS and increased the activity of SOD in ox-LDL-induced HUVECs, and these effects were largely alleviated by the addition of the miR-186-5p inhibitor (Figures 4H–J). Circ_0068087 silencing promoted the proliferation of ox-LDL-induced HUVECs, and the addition of the miR-186-5p inhibitor restrained cell proliferation again (Supplementary Figure 3A). In addition, we found that circ_0068087 knockdown facilitated cell migration, and cell migration was suppressed in the si-circ_0068087 and miR-186-5p inhibitor co-transfected group in ox-LDL-induced HUVECs (Supplementary Figure 3B). Taken together, ox-LDL induced the injury of HUVECs partly through upregulating circ_0068087 and downregulating miR-186-5p.

### MiR-186-5p Interacts With the 3′UTR of ROBO1 in HUVECs

**Figure 5:**
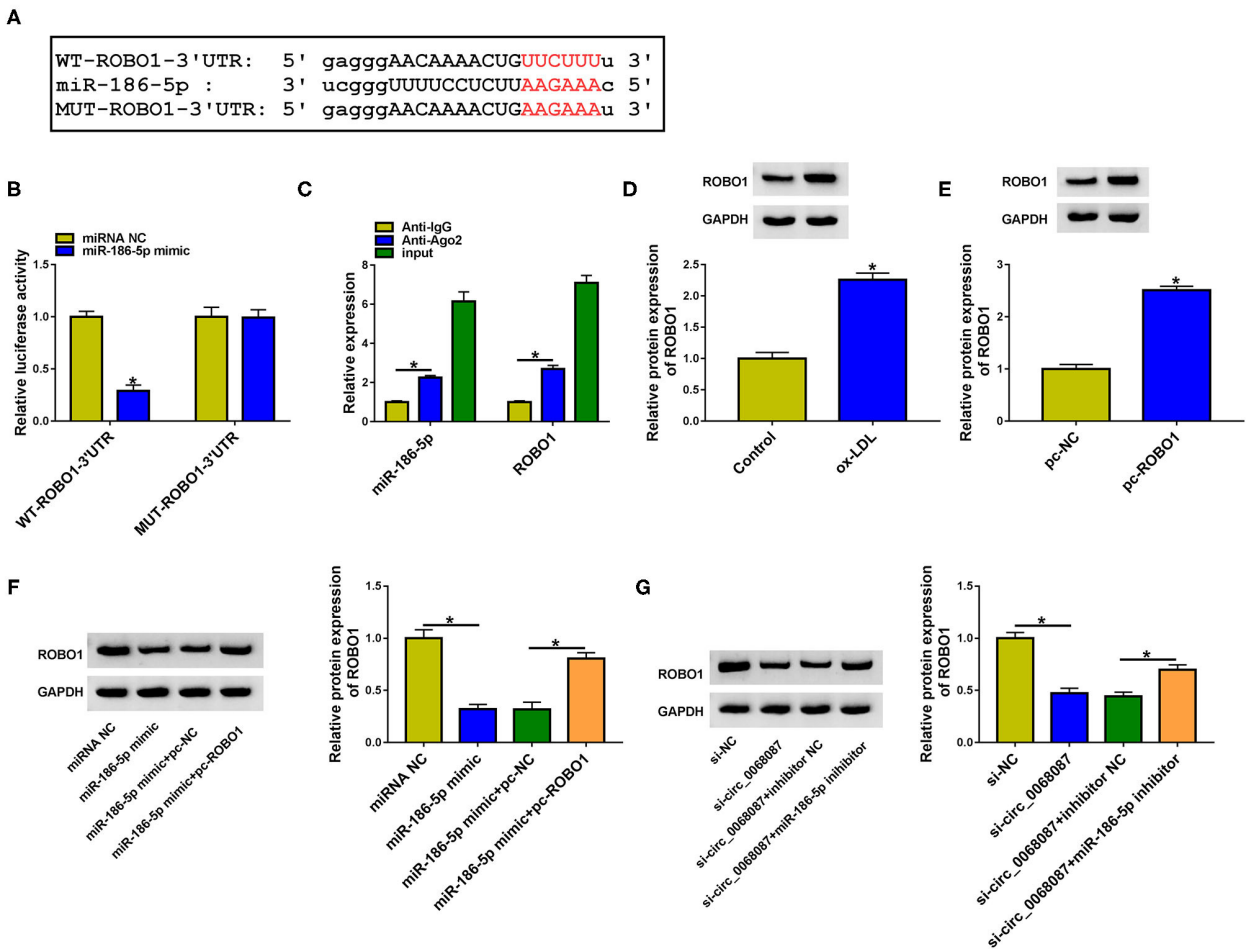
MiR-186-5p interacts with the 3'UTR of ROBO1 in HUVECs. **(A)** The putative binding sequence between miR-186-5p and ROBO1 predicted by the StarBase database is shown. **(B)** Dual-luciferase reporter assay was performed to confirm if ROBO1 was a target of miR-186-5p in HUVECs. This experiment was performed three times with three technical repetitions each time. An unpaired Student *t*-test was used to evaluate the differences. **(C)** RIP assay was applied to confirm the target relation between ROBO1 and miR-186-5p. This experiment was performed three times with three technical repetitions each time. An unpaired Student *t*-test was used to evaluate the differences. **(D)** The protein expression of ROBO1 was measured in HUVECs treated with ox-LDL (40 mg/L, 24 h) by Western blot assay. This experiment was performed three times. An unpaired Student *t*-test was used to evaluate the differences. **(E)** The overexpression efficiency of ROBO1 plasmid (pc-ROBO1) was analyzed by Western blot assay. This experiment was performed three times. An unpaired Student *t*-test was used to evaluate the differences. **(F)** HUVECs were transfected with miR-186-5p mimic alone or together with pc-ROBO1, and the protein level of ROBO1 was measured by Western blot assay. This experiment was performed three times. One-way ANOVA followed by Tukey's *post hoc* test was used to assess the differences. **(G)** The protein expression of ROBO1 was determined in HUVECs transfected with si-circ_0068087 alone or together with miR-186-5p inhibitor by Western blot assay. This experiment was performed three times. One-way ANOVA followed by Tukey's *post hoc* test was used to assess the differences. *P < 0.05.

We used bioinformatic software StarBase to predict the downstream targets of miR-186-5p. Among all the predicted mRNA targets of miR-186-5p, we focused on five mRNAs due to their vital roles in AS progression, including ROBO1 (16), KLF4 (25), STAT3 (26), AURKA (27), and IFI44L (28). ROBO1 was selected for further analysis due to it having the most significant negative regulatory relationship with miR-186-5p (Supplementary Figure 2B). The putative binding sites between miR-186-5p and ROBO1 are shown in Figure 5A. Subsequently, the target interaction between miR-186-5p and ROBO1 was validated by the dual-luciferase reporter assay. Luciferase activity of wild-type plasmid (WT-ROBO1-3'UTR) was markedly decreased by the transfection of miR-186-5p mimic rather than miRNA NC (Figure 5B), suggesting that ROBO1 was a target of miR-186-5p in HUVECs. The miR-186-5p-binding sites in ROBO1 3'UTR were mutated to “AAGAAA,” and the luciferase activity of mutant plasmid (MUT-ROBO1-3'UTR) was unchanged by the transfection of miR-186-5p mimic or miRNA NC (Figure 5B), demonstrating that miR-186-5p bound to the 3'UTR of ROBO1 via the predicted sites. The binding relation between miR-186-5p and ROBO1 was also confirmed by RIP assay (Figure 5C). ox-LDL treatment increased the protein level of ROBO1 in HUVECs (Figure 5D). High transfection efficiency of ROBO1 plasmid (pc-ROBO1) was verified by Western blot assay (Figure 5E). MiR-186-5p overexpression reduced ROBO1 protein expression, and the protein level of ROBO1 was largely rescued by the addition of ROBO1 plasmid in HUVECs (Figure 5F). HUVECs were cotransfected with si-circ_0068087 and miR-186-5p inhibitor to analyze the regulation among circ_0068087, miR-186-5p, and ROBO1. As shown in Figure 5G, circ_0068087 knockdown reduced the protein expression of ROBO1, and the silence of miR-186-5p largely rescued the protein level of ROBO1 in HUVECs. These results suggest that ROBO1 was a target of miR-186-5p, and ROBO1 was regulated by circ_0068087/miR-186-5p axis in HUVECs.

### ROBO1 Overexpression Largely Overturns miR-186-5p-Mediated Effects in HUVECs Upon ox-LDL Exposure

**Figure 6:**
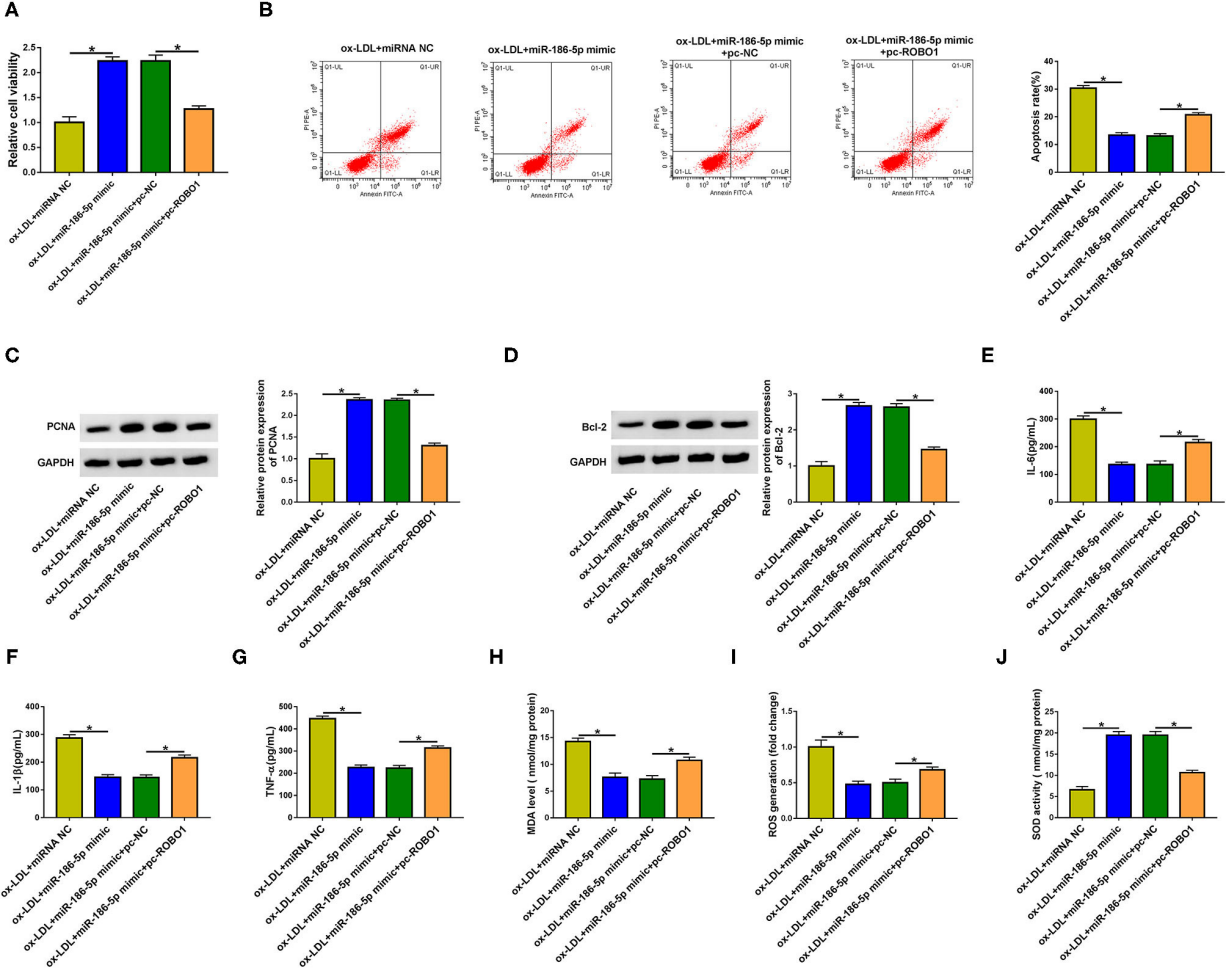
ROBO1 overexpression largely overturns miR-186-5p-mediated effects in HUVECs upon ox-LDL exposure. **(A–J)** HUVECs transfected with miR-186-5p mimic alone or together with pc-ROBO1 were stimulated with ox-LDL (40 mg/L, 24 h). One-way ANOVA followed by Tukey's *post hoc* test was used to assess the differences. **(A)** Cell viability was assessed by MTT assay. This experiment was performed three times with five technical repetitions each time. **(B)** The apoptosis rate was measured by flow cytometry. This experiment was performed three times with three technical repetitions each time. **(C)** The level of PCNA protein was measured by Western blot assay. This experiment was performed three times. **(D)** Western blot assay was utilized to detect the protein level of Bcl-2 in HUVECs. This experiment was performed three times. **(E–G)** The levels of IL-6, IL-1β, and TNF-α were determined by ELISA. This experiment was performed three times with three technical repetitions each time. **(H–J)** Cell oxidative stress was analyzed by corresponding kits. This experiment was performed three times with three technical repetitions each time. *P < 0.05.

HUVECs were transfected with miR-186-5p mimic alone or together with pc-ROBO1 prior to ox-LDL exposure to conduct rescue experiments. MiR-186-5p overexpression elevated cell viability and suppressed cell apoptosis, inflammation, and oxidative stress (Figures 6A–J), which further demonstrated that miR-186-5p protected HUVECs against ox-LDL-induced injury. The overexpression of ROBO1 suppressed cell viability in miR-186-5p-overexpressed HUVECs upon ox-LDL exposure (Figure 6A). Cell apoptosis was induced again in miR-186-5p and pc-ROBO1 cotransfected group in ox-LDL-exposed HUVECs (Figure 6B). MiR-186-5p overexpression-mediated upregulation in the expression of PCNA, Ki67, and Bcl-2 and downregulation in the level of Bax were largely reversed by the accumulation of ROBO1 in ox-LDL-stimulated HUVECs (Figures 6C,D, Supplementary Figure 1C), demonstrating that miR-186-5p protected HUVECs from an ox-LDL-induced suppressive effect on cell viability and promoting effect on cell apoptosis partly by reducing ROBO1 expression. The overexpression of ROBO1 largely rescued the release of inflammatory cytokines (IL-6, IL-1β, and TNF-α) in miR-186-5p-overexpressed HUVECs upon ox-LDL exposure (Figures 6E–G). MiR-186-5p overexpression reduced the levels of MDA and ROS and increased the activity of SOD although these effects were all counteracted by the overexpression of ROBO1 in ox-LDL-induced HUVECs (Figures 6H–J). Taken together, miR-186-5p protected HUVECs from ox-LDL-induced damage partly by reducing the ROBO1 level.

Vascular cellular adhesion molecule-1 (VCAM-1) and intracellular adhesion molecule-1 (ICAM-1) exert vital roles in recruiting inflammatory monocytes into the vascular wall and initiating AS (29). Based on former articles (30), ox-LDL induced the production of ICAM-1 and VCAM-1. We explored the role of the circ_0068087/miR-186-5p/ROBO1 axis in regulating the levels of VCAM-1 and ICAM-1 in HUVECs. Circ_0068087 interference reduced the levels of VCAM-1 and ICAM-1, and the overexpression of ROBO1 rescued the expression of VCAM-1 and ICAM-1 in ox-LDL-induced HUVECs (Supplementary Figures 4A,B).

## Discussion

AS induced by multiple vasculopathy contributes to severe cardiovascular disorders. Endothelial cell injury theory considers artery atheromatous plaque as the product of endothelial injury (31), and ox-LDL is an important risk factor that induces the dysfunction of endothelial cells. In the current study, we explored the role of circ_0068087 in AS progression using ox-LDL-induced AS cell model.

CircRNAs are a class of non-coding transcripts featuring a stable and covalently closed circular structure (32). Accumulating studies associate the functions of circRNAs with CVDs, including ischemia reperfusion injury, cardiac fibrosis, and AS (7, 33). For instance, circ-SATB2 modulates the phenotypic differentiation, proliferation, apoptosis, and migration of vascular smooth muscle cells by targeting the miR-939/STIM1 axis (34). Circ-PTPRA contributes to AS development through elevating SP1 expression via sponging miR-636 (35). Cheng et al. demonstrate that circ_0068087 is upregulated upon high glucose treatment, and it promotes the inflammation and dysfunction of HUVECs by sponging miR-197 in diabetes mellitus (10). However, its biological role in AS has never been explored. Here, we analyzed the function of circ_0068087 in AS progression using ox-LDL-induced AS cell model. We found that ox-LDL stimulation upregulated the expression of circ_0068087. ox-LDL-induced injury in HUVECs was largely reversed by the silence of circ_0068087, suggesting that the upregulation of circ_0068087 was essential for ox-LDL-induced dysfunction in HUVECs.

CircRNAs can act as miRNA sponges to function in human diseases. For instance, circ_33186 facilitates the development of osteoarthritis by acting as miR-127-5p sponge (36). Circ-HIPK3 promotes the proliferation and motility of colorectal cancer cells by absorbing miR-7 (37). We identified miR-186-5p as a target of circ_0068087 in HUVECs. Hypoxia is reported to downregulate miR-186 expression, and circ_0010729 promotes the proliferation and inhibits the apoptosis of high glucose-induced HUVECs by targeting miR-186/HIF-1α axis (13). Nevertheless, the biological role of miR-186-5p in regulating ox-LDL-induced injury of HUVECs has never been illustrated. We found that ox-LDL stimulation reduced miR-186-5p level in HUEVCs. In addition, miR-186-5p was negatively regulated by circ_0068087 in HUVECs. Through rescue experiments, we found that circ_0068087 silencing attenuated ox-LDL-induced dysfunction of endothelial cells partly by upregulating miR-186-5p.

MiRNAs regulate cellular physiological and pathological processes through inducing translational repression or degradation of mRNAs via binding to them (11). For example, miR-182 attenuates the progression of cyanotic congenital heart disorder by downregulating the expression of HES1 (38). MiR-210 silencing contributes to pancreatic cancer progression by upregulating the level of E2F3 (39). ROBO1 was validated as a downstream target of miR-186-5p in HUVECs. Pan et al. find that ROBO1 is upregulated by high glucose treatment in HUVECs (16). ox-LDL exposure upregulated the protein level of ROBO1 in HUVECs. Furthermore, we find that ROBO1 was reverse modulated by miR-186-5p. Circ_0068087 positively regulated ROBO1 expression by acting as an miR-186-5p sponge in HUVECs. Through compensation experiments, we found that miR-186-5p exhibited a protective role in ox-LDL-induced HUVECs partly by downregulating ROBO1.

In the future, we will further explore the working mechanism of ROBO1 in regulating ox-LDL-induced changes in the cellular behaviors of HUVECs. In addition, the *in vivo* function of circ_0068087/miR-186-5p/ROBO1 axis in AS progression needs to be confirmed.

**Figure 7:**
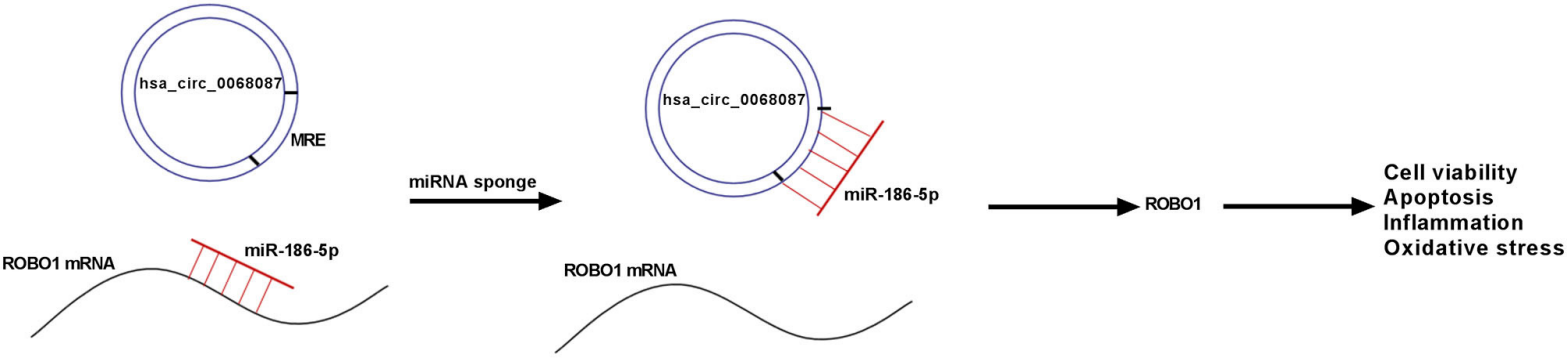
Diagram of the working mechanism of circ_0068087 in ox-LDL-induced HUVECs.

Taken together, circ_0068087 aggravated ox-LDL-mediated dysfunction in HUVECs by sponging miR-186-5p via its miRNA response element (MRE), thereby upregulating ROBO1 (Figure 7). Our study provides a profound understanding about the function of circ_0068087 in AS and novel potential therapeutic targets for AS.

## Data Availability Statement

The raw data supporting the conclusions of this article will be made available by the authors, without undue reservation.

## Author Contributions

SL had full access to all of the data in the study and takes responsibility for the integrity of the data and the accuracy of the data analysis. LQ and LY: acquisition of data. SL and TH: study concept and design. TH, LQ, and LY: critical revision of the manuscript for important intellectual content. SL: administrative, technical, or material support, and study supervision. All authors contributed to the article and approved the submitted version.

## Conflict of Interest

The authors declare that the research was conducted in the absence of any commercial or financial relationships that could be construed as a potential conflict of interest.

## Supplementary Material

Supplementary Figure 1 The regulatory role of circ_0068087/miR-186-5p/ROBO1 axis on the protein expression of proliferation marker Ki67 and pro-apoptotic protein Bax in ox-LDL-induced HUVECs. **(A)** Western blot assay was adopted to analyze the protein levels of Ki67 and Bax in HUVECs in the following four groups: control, ox-LDL, ox-LDL + si-NC, and ox-LDL + si-circ_0068087. This experiment was performed three times. One-way ANOVA followed by Tukey's *post hoc* test was used to assess the differences. **(B)** The protein expression of Ki67 and Bax in ox-LDL-induced HUVECs transfected with si-circ_0068087 alone or together with miR-186-5p inhibitor was determined by Western blot assay. This experiment was performed three times. One-way ANOVA followed by Tukey's *post hoc* test was used to assess the differences. **(C)** The protein expression of Ki67 and Bax was examined in ox-LDL-induced HUVECs transfected with miR-186-5p mimic alone or together with pc-ROBO1 by Western blot assay. This experiment was performed three times. One-way ANOVA followed by Tukey's *post hoc* test was used to assess the differences. ^*^*P* < 0.05.

Supplementary Figure 2 The candidate targets of circ_0068087 and miR-186-5p predicted by bioinformatic databases. **(A)** Two bioinformatic databases (circinteractome and circBank) were utilized to predict the possible miRNA targets of circ_0068087. The expression of miR-186-5p, miR-140-3p, miR-640, miR-515-5p and miR-665 was determined in HUVECs transfected with si-NC or si-circ_0068087 by RT-qPCR. This experiment was performed three times with three technical repetitions each time. An unpaired Student *t*-test was used to evaluate the differences. **(B)** The possible mRNA targets of miR-186-5p were predicted by the StarBase database. RT-qPCR was applied to analyze the expression of five candidate mRNA targets of miR-186-5p in HUVECs transfected with inhibitor NC or miR-186-5p inhibitor. This experiment was performed three times with three technical repetitions each time. An unpaired Student *t*-test was used to evaluate the differences.^*^*P* < 0.05.

Supplementary Figure 3 Circ_0068087 knockdown promotes the proliferation and migration of HUVECs partly through upregulating miR-186-5p. **(A)** MTT assay was applied to analyze cell proliferation ability in HUVECs transfected with si-circ_0068087 alone or together with miR-186-5p inhibitor prior to ox-LDL exposure. This experiment was performed three times with five technical repetitions each time. One-way ANOVA followed by Tukey's *post hoc* test was used to assess the differences. **(B)** Transwell migration assay was utilized to analyze cell migration ability. This experiment was performed three times with three technical repetitions each time. One-way ANOVA followed by Tukey's *post hoc* test was used to assess the differences. ^*^*P* < 0.05.

Supplementary Figure 4 Circ_0068087 silencing reduces the expression of ICAM-1 and VCAM-1 in ox-LDL-induced HUVECs partly through downregulating ROBO1. **(A,B)** HUVECs were introduced with si-circ_0068087 alone or together with pc-ROBO1 prior to ox-LDL exposure. The mRNA expression of ICAM-1 and VCAM-1 was determined in HUVECs by RT-qPCR. This experiment was performed three times with three technical repetitions each time. One-way ANOVA followed by Tukey's *post hoc* test was used to assess the differences. ^*^*P* < 0.05.
