# Supplementary figures and images for: Circ_0068087 Silencing Ameliorates Oxidized Low-Density Lipoprotein-Induced Dysfunction in Vascular Endothelial Cells Depending on miR-186-5p-Mediated Regulation of Roundabout Guidance Receptor 1
Source: Front Cardiovasc Med. 2021 May 26;8:650374. doi: 10.3389/fcvm.2021.650374 (PMC8187595; doi:10.3389/fcvm.2021.650374)

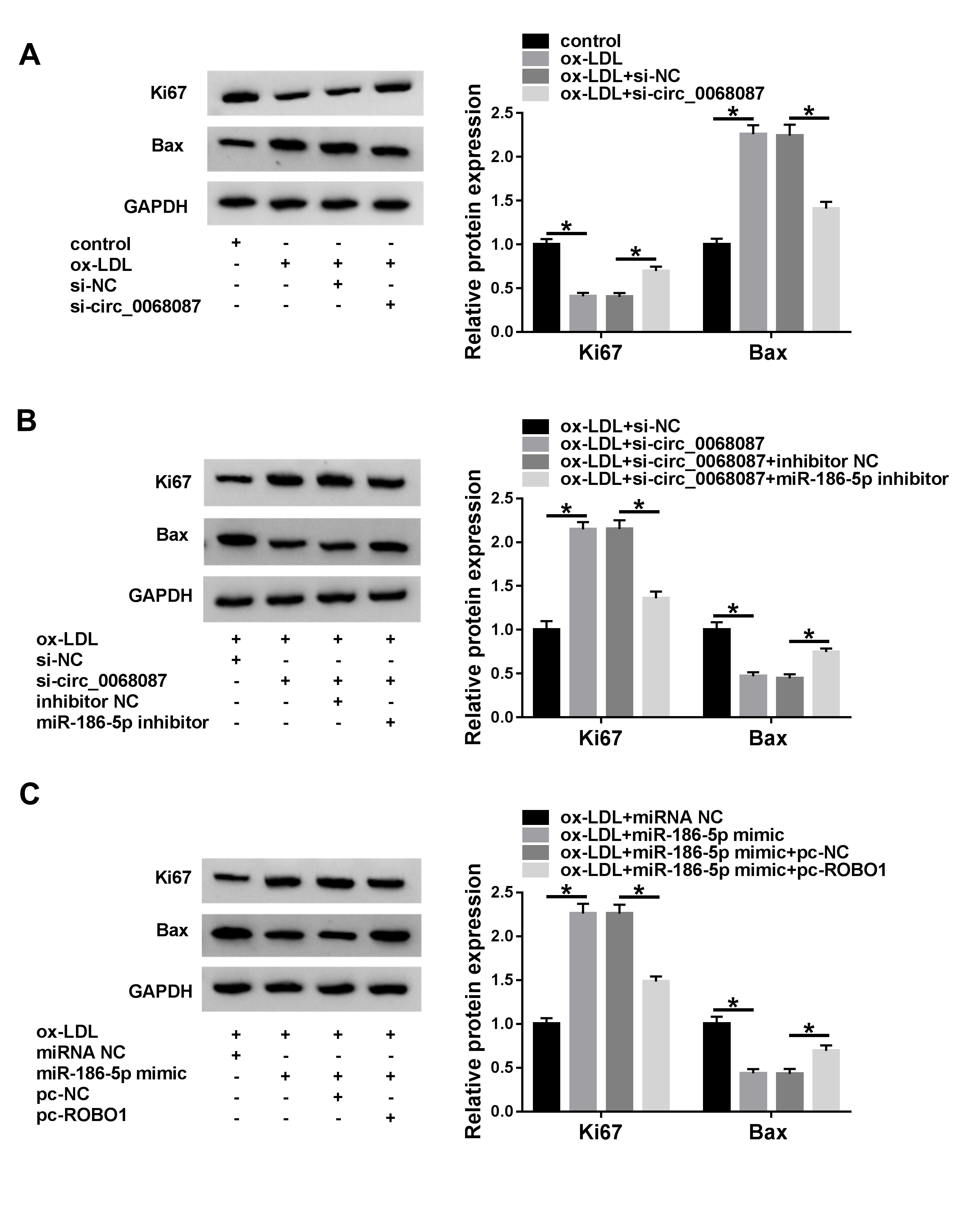

Supplement: Supplementary Figure 1 — The regulatory role of circ_0068087/miR-186-5p/ROBO1 axis on the protein expression of proliferation marker Ki67 and pro-apoptotic protein Bax in ox-LDL-induced HUVECs. (A) Western blot assay was adopted to analyze the protein levels of Ki67 and Bax in HUVECs in the following four groups: control, ox-LDL, ox-LDL + si-NC, and ox-LDL + si-circ_0068087. This experiment was performed three times. One-way ANOVA followed by Tukey's post hoc test was used to assess the differences. (B) The protein expression of Ki67 and Bax in ox-LDL-induced HUVECs transfected with si-circ_0068087 alone or together with miR-186-5p inhibitor was determined by Western blot assay. This experiment was performed three times. One-way ANOVA followed by Tukey's post hoc test was used to assess the differences. (C) The protein expression of Ki67 and Bax was examined in ox-LDL-induced HUVECs transfected with miR-186-5p mimic alone or together with pc-ROBO1 by Western blot assay. This experiment was performed three times. One-way ANOVA followed by Tukey's post hoc test was used to assess the differences. *P < 0.05. [file Image_1.TIF]

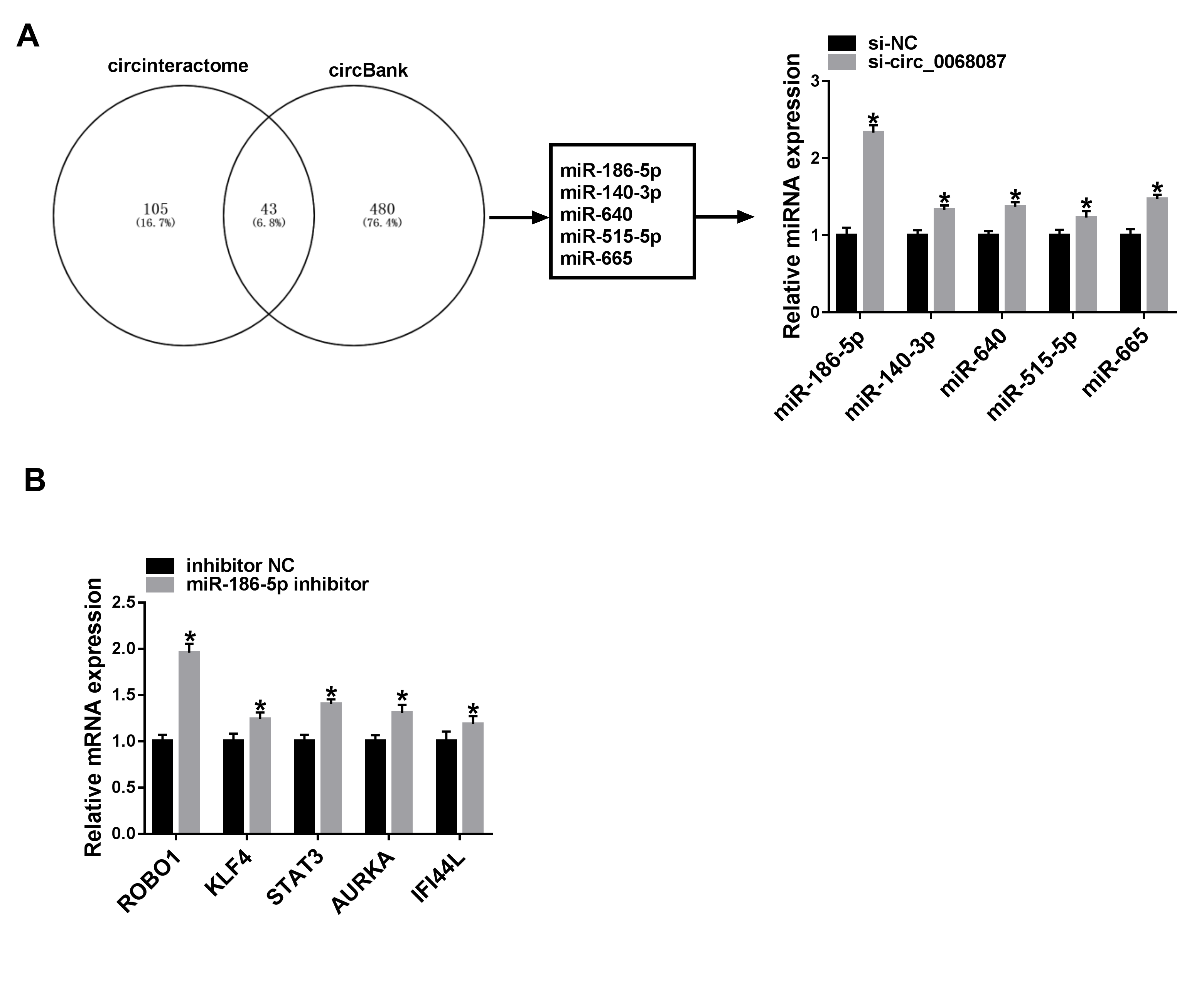

Supplement: Supplementary Figure 2 — The candidate targets of circ_0068087 and miR-186-5p predicted by bioinformatic databases. (A) Two bioinformatic databases (circinteractome and circBank) were utilized to predict the possible miRNA targets of circ_0068087. The expression of miR-186-5p, miR-140-3p, miR-640, miR-515-5p and miR-665 was determined in HUVECs transfected with si-NC or si-circ_0068087 by RT-qPCR. This experiment was performed three times with three technical repetitions each time. An unpaired Student t-test was used to evaluate the differences. (B) The possible mRNA targets of miR-186-5p were predicted by the StarBase database. RT-qPCR was applied to analyze the expression of five candidate mRNA targets of miR-186-5p in HUVECs transfected with inhibitor NC or miR-186-5p inhibitor. This experiment was performed three times with three technical repetitions each time. An unpaired Student t-test was used to evaluate the differences.*P < 0.05. [file Image_2.TIF]

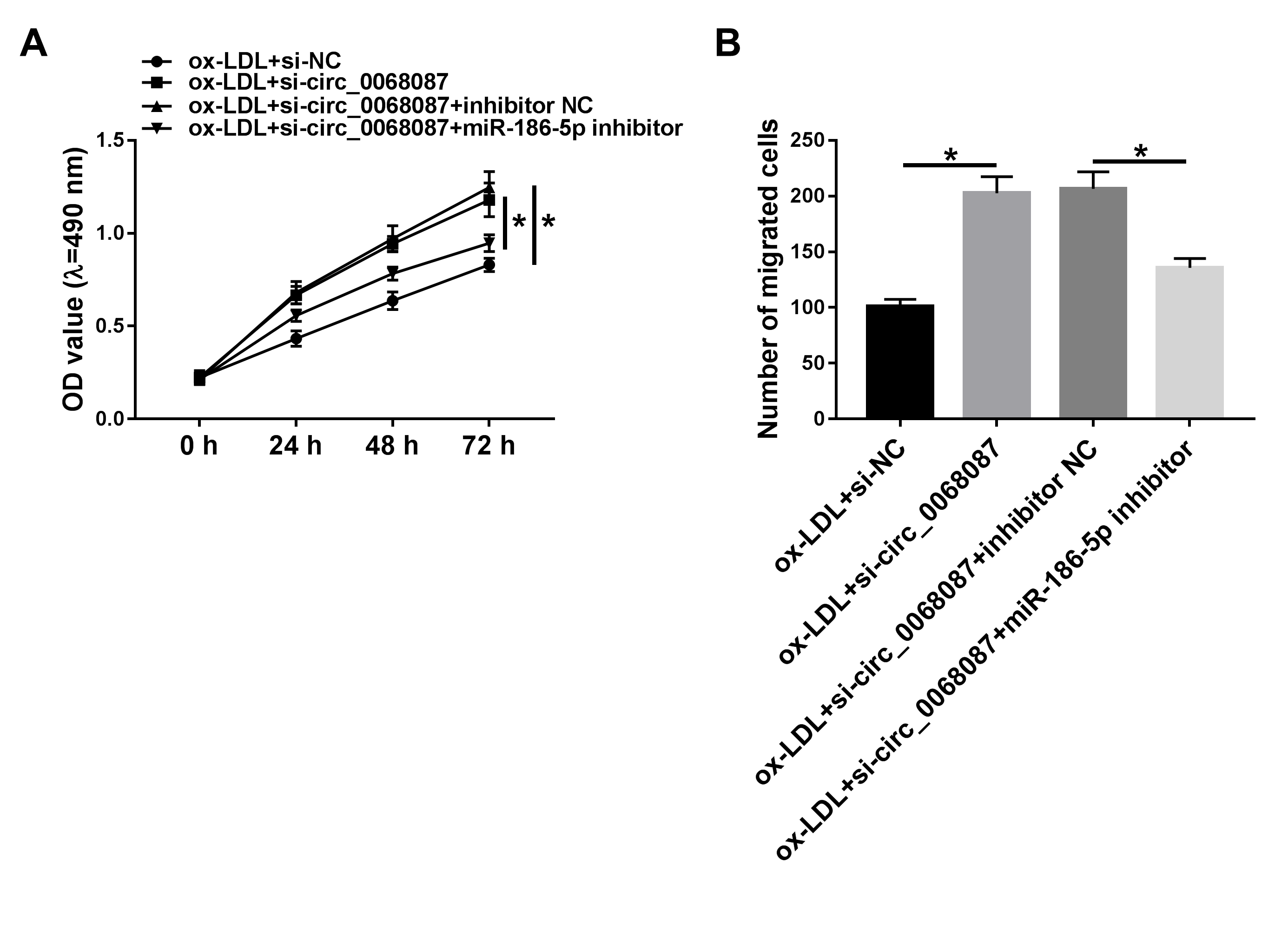

Supplement: Supplementary Figure 3 — Circ_0068087 knockdown promotes the proliferation and migration of HUVECs partly through upregulating miR-186-5p. (A) MTT assay was applied to analyze cell proliferation ability in HUVECs transfected with si-circ_0068087 alone or together with miR-186-5p inhibitor prior to ox-LDL exposure. This experiment was performed three times with five technical repetitions each time. One-way ANOVA followed by Tukey's post hoc test was used to assess the differences. (B) Transwell migration assay was utilized to analyze cell migration ability. This experiment was performed three times with three technical repetitions each time. One-way ANOVA followed by Tukey's post hoc test was used to assess the differences. *P < 0.05. [file Image_3.TIF]

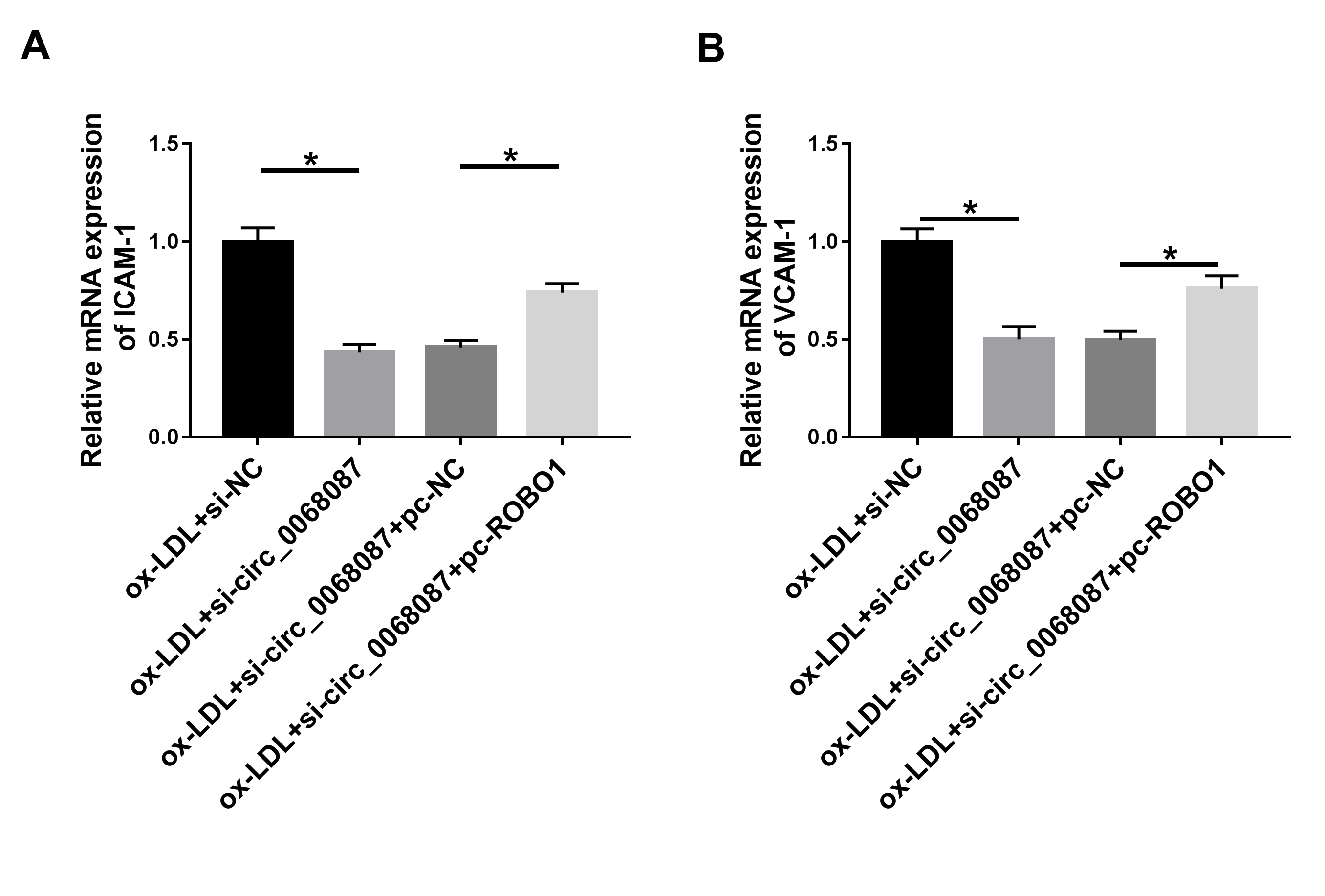

Supplement: Supplementary Figure 4 — Circ_0068087 silencing reduces the expression of ICAM-1 and VCAM-1 in ox-LDL-induced HUVECs partly through downregulating ROBO1. (A,B) HUVECs were introduced with si-circ_0068087 alone or together with pc-ROBO1 prior to ox-LDL exposure. The mRNA expression of ICAM-1 and VCAM-1 was determined in HUVECs by RT-qPCR. This experiment was performed three times with three technical repetitions each time. One-way ANOVA followed by Tukey's post hoc test was used to assess the differences. *P < 0.05. [file Image_4.TIF]
